# MUC1 alleviates PM2.5-induced airway inflammation by inhibiting the IRAK4/NF-κB/NLRP3 mediated pyroptosis in airway epithelial cells

**DOI:** 10.3389/fimmu.2025.1653184

**Published:** 2025-10-16

**Authors:** You Zhou, Kai Yang, Jian Wang, Ruijuan Guan, Wei Liu, Chaowei Wen, Liang Yuan, Tiange Zhang, Wei Feng, Yuanyuan Li, Jingyi Xu, Wenju Lu

**Affiliations:** ^1^ Department of Pulmonary and Critical Care Medicine, Guangdong Provincial Hospital of Chinese Medicine, Guangzhou, Guangdong, China; ^2^ State Key Laboratory of Respiratory Disease, National Clinical Research Center for Respiratory Disease, Guangzhou Institute of Respiratory Health, The First Affiliated Hospital of Guangzhou Medical University, Guangzhou, Guangdong, China; ^3^ Guangzhou University of Chinese Medicine, Guangzhou, Guangdong, China

**Keywords:** PM2.5, mucin 1, airway inflammation, pyroptosis, irak4

## Abstract

**Backgrounds:**

Exposure to fine particulate matter (PM2.5) triggers airway inflammation through the activation of the Toll-like receptor 4 (TLR4)/nuclear factor-κB (NF-κB) signaling pathway, contributing to the pathogenesis of various acute and chronic respiratory diseases. Mucin 1 (MUC1), a transmembrane glycoprotein highly expressed on airway epithelial cells, is known to modulate TLR-mediated inflammatory responses. However, the specific role and molecular mechanism by which MUC1 regulates PM2.5-induced airway inflammation remain inadequately understood.

**Methods:**

Lung injury models were established using Muc1^+/+^ and Muc1^-/-^ rats via intranasal instillation of PM2.5. Histopathological changes and inflammatory responses were evaluated following treatment. In parallel, human bronchial epithelial cells (16HBE) were transfected with MUC1 overexpression and knockdown plasmids, along with corresponding controls. An IRAK4-specific inhibitor was employed to explore the mechanistic role of MUC1 in regulating the TLR4/IRAK4/NF-κB signaling cascade and associated pyroptosis.

**Results:**

PM2.5 exposure caused notable epithelial disruption, inflammatory infiltration, and submucosal fibrosis in Muc1^-/-^ rats compared to Muc1^+/+^ controls. While TLR4 expression was not significantly altered, NF-κB and NLRP3 inflammasome activity were markedly elevated in Muc1-deficient rats. In 16HBE cells, MUC1 overexpression attenuated, whereas MUC1 knockdown exacerbated, PM2.5-induced activation of the IRAK4/NF-κB signaling axis and pyroptosis-related markers (IL-1β, IL-18, NLRP3, GSDMD). Furthermore, pharmacological inhibition of IRAK4 mitigated these effects, confirming the involvement of the IRAK4 pathway in MUC1-mediated protection.

**Conclusions:**

MUC1 is upregulated in airway epithelial cells upon PM2.5 exposure and serves as a key endogenous inhibitor of airway inflammation. It exerts its anti-inflammatory and anti-pyroptotic effects by suppressing IRAK4 phosphorylation, thereby modulating downstream NF-κB/NLRP3/GSDMD signaling. These findings highlight MUC1 as a potential therapeutic target for PM2.5-induced airway inflammatory diseases.

## 1 Intoduction

Air pollution has emerged as a major global public health concern, with increasing evidence linking fine particulate matter (PM), particularly those with an aerodynamic diameter ≤2.5 μm (PM2.5), to adverse health outcomes (1). Due to its small size and large specific surface area, PM2.5 can adsorb toxic substances such as heavy metals, bacteria, and viruses (2). These particles remain airborne for extended periods, penetrate deeply into the respiratory tract, deposit in bronchi and alveoli, and may even enter the bloodstream, leading to chronic and sustained effects that elevate the risk of various respiratory diseases (3). Epidemiological studies have consistently demonstrated that PM2.5 exposure contributes to the development of airway inflammatory disorders, including allergic airway diseases, acute respiratory infections, and chronic obstructive pulmonary disease (COPD) (4). Therefore, elucidating the mechanisms by which PM2.5 induces airway inflammation is of critical importance.

Pyroptosis, a form of programmed inflammatory cell death, plays a pivotal role in the innate immune response and the pathogenesis of inflammation (5). In mammalian cells, classical pyroptosis pathways are typically triggered by microbial components such as bacterial lipopolysaccharide (LPS), which bind to toll-like receptor 4 (TLR4), promote nuclear factor-kappa B (NF-κB) translocation to the nucleus, and activate the NOD-like receptor protein 3 (NLRP3) inflammasome. Activation of this inflammasome results in Gasdermin D (GSDMD) cleavage, leading to membrane pore formation, cell lysis, and the release of inflammatory mediators. Simultaneously, active caspase-1 processes pro-interleukin-1β (IL-1β) and pro-interleukin-18 (IL-18), enhancing inflammatory cell recruitment and amplifying the inflammatory response (6). Airway epithelial cells, which form the structural and functional barrier of the airway wall, have been shown to undergo pyroptosis in mouse models of toluene diisocyanate-induced asthma (7). Our previous work also demonstrated that PM2.5 triggers inflammatory responses via activation of the TLR4/NF-κB signaling axis (8). However, whether PM2.5 induces pyroptosis in airway epithelial cells to promote airway inflammation remains to be fully elucidated.

Mucin 1 (MUC1), the first identified mucin gene, encodes a type I transmembrane glycoprotein expressed in humans and animals. It is widely distributed on the epithelial surfaces of the respiratory, gastrointestinal, and reproductive tracts, as well as in various immune-related cells (9). MUC1 is abundantly expressed on the surface of airway epithelial cells (10) and has been identified as an important endogenous anti-inflammatory mediator during airway inflammation (11). Several studies have shown that MUC1 suppresses inflammation by interfering with TLR-mediated signaling pathways (12, 13). Interleukin-1 receptor-associated kinase 4 (IRAK4), a serine/threonine kinase, is a key downstream effector in TLR signaling and plays a central role in mediating systemic inflammatory responses (14, 15). Evidence from gastric inflammation models indicates that MUC1 can inhibit IRAK4 activation and suppress NF-κB signaling, thereby downregulating the transcription of NLRP3 and other inflammatory mediators (16). In the context of PM2.5 exposure, TLR4 has been identified as the primary receptor mediating the inflammatory response, with the silicon (Si) component of PM2.5 potentially forming hydrolyzed complexes (e.g., Si-OH) that are recognized by TLR4 (17–19). However, it remains unclear whether MUC1 modulates IRAK4 activity in this context and how this interaction affects downstream pyroptosis and inflammation in airway epithelial cells.

Based on this background, we hypothesized that MUC1 alleviates PM2.5-induced airway inflammation by targeting IRAK4 to inhibit the TLR4/NF-κB signaling pathway and suppress NLRP3 activation, thereby preventing pyroptosis of airway epithelial cells. To test this hypothesis, we employed Muc1^+/+^ and Muc1^−/−^ rats subjected to intranasal PM2.5 instillation to model airway inflammation *in vivo*. In parallel, human bronchial epithelial 16HBE cells were transfected with MUC1 overexpression or knockdown plasmids, with or without IRAK4 inhibitor treatment, to evaluate pyroptosis and inflammatory responses *in vitro*. This study provides a comprehensive evaluation of the regulatory role of MUC1 in PM2.5-induced airway inflammation and offers new theoretical insights into potential therapeutic strategies for PM2.5-related respiratory diseases.

## 2 Materials and methods

### 2.1 Chemicals and reagents

Standard Reference Material 1649b Urban Dust (SRM 1649b), representing PM2.5, was obtained from the National Institute of Standards and Technology (NIST, Gaithersburg, MD, USA). The following primary antibodies were used in this study: anti-TLR4 (#WL00196, Wanleibio, China); anti-MUC1 (A19081, ABclonal, China); anti-IRAK4 (#YM0380, Immunoway, USA); anti-phospho-IRAK4 (#DF7567, Affinity, China); anti-NF-κB p65 (#AF5006, Affinity, China); anti-GSDMD (#A18281, ABclonal, China); anti-NLRP3 (#A12694, Abclonal, China); anti-ASC (#sc-514414, Santa Cruz Biotechnology, USA); and anti-Caspase-1 (#sc-392736, Santa Cruz Biotechnology, USA). Secondary antibodies included Cy3-conjugated goat anti-rabbit IgG (#A27039, Invitrogen, USA) and FITC-conjugated goat anti-mouse IgG (#ab6785, Abcam, UK). All cell culture reagents were supplied by Gibco (Carlsbad, CA, USA).

The certified reference material of SRM 1649b is fully characterized, with its precise chemical composition and particle size distribution detailed in the NIST Certificate of Analysis. Major constituents comprise polycyclic aromatic hydrocarbons (PAHs), nitro-PAHs, inorganic elements, and carbon fractions. The majority of particles are distributed within the fine particulate range (aerodynamic diameter ≤ 2.5 μm). Employing this standardized material enhances experimental reproducibility and enables reliable cross-study comparisons of PM2.5-related biological responses.

### 2.2 Animals and treatments

All animal experiments were conducted in accordance with the ethical guidelines approved by the Ethics Committee of Guangzhou Medical University (Approval No. 2021085). Male Sprague-Dawley rats (6–8 weeks old) with Muc1 wild-type (Muc1^+/+^) and knockout (Muc1^−/−^) backgrounds, generously provided by Prof. Kim KC (University of Arizona, USA), were housed under specific pathogen-free (SPF) conditions at a constant temperature of 24°C and a 12-hour light/dark cycle, with unrestricted access to food and water. The animals were randomly assigned to four experimental groups: (1) Muc1^+/+^ rats receiving intranasal instillation of sterile water (Control-Muc1^+/+^); (2) Muc1^+/+^ rats instilled with PM2.5 (PM2.5-Muc1^+/+^); (3) Muc1^−/−^ rats instilled with sterile water (Control-Muc1^−/−^); and (4) Muc1^−/−^ rats instilled with PM2.5 (PM2.5-Muc1^−/−^). To induce airway inflammation, rats in the PM2.5-exposed groups were administered PM2.5 suspension (0.5 mg/mL, 100 μL/rat) via intranasal instillation on days 1, 7, and 14. The selection of the 0.5 mg/mL PM2.5 concentration was based on our previous dose–response studies, which demonstrated that this dose effectively induces significant airway inflammation and pathological injury without causing excessive mortality, as observed in our established rat model of acute lung injury (20). Furthermore, this concentration has been validated to specifically activate the TLR4/NF-κB signaling pathway, as evidenced by its responsiveness to pharmacological inhibition in prior work (8), thereby supporting its relevance for investigating pathway-specific inflammatory responses rather than nonspecific cytotoxicity. Control groups received an equivalent volume of sterile water. All rats were sacrificed within 24 hours following the final instillation.

### 2.3 Cell culture

Human bronchial epithelial cells (16HBE) were procured from the Cell Bank of the Chinese Academy of Sciences (Shanghai, China) and cultured in Dulbecco’s Modified Eagle Medium (DMEM) supplemented with 10% fetal bovine serum (FBS), 100 KU/L penicillin, and 100 mg/L streptomycin. Cells were maintained in a humidified incubator at 37 °C with 5% CO_2_. PM2.5 suspension (0.5 mg/mL) was prepared in DMEM medium. For genetic manipulation, 16HBE cells were transfected with either a MUC1 overexpression plasmid (pcDNA3.1-MUC1), a MUC1 knockdown construct (sh-MUC1), or their respective control vectors (pcDNA3.1 from FenghuiBio, China; pRNA-H1.1 from Wanleibio, China) using Lipofectamine 3000 (Invitrogen). The efficiency of transfection and the consequent modulation of MUC1 expression were assessed by RT-qPCR and Western blot, which confirmed significant alterations at the mRNA and protein levels, respectively. Forty-eight hours post-transfection, cells were exposed to either PM2.5 suspension or an equivalent volume of DMEM for an additional 24 hours. Where indicated, IRAK4 activity was pharmacologically inhibited by pretreatment with 1 μM IRAK1/4 Inhibitor I (MCE) 45 minutes prior to PM2.5 stimulation. The PM2.5 concentration of 0.5 mg/mL used *in vitro* was selected to maintain consistency with our *in vivo* model and has been previously shown to elicit robust inflammatory and pyroptotic responses in airway epithelial cells without inducing overwhelming cell death, thereby allowing for the evaluation of specific molecular pathways such as TLR4/NF-κB and NLRP3 inflammasome activation.

### 2.4 Preparation of PM2.5 suspension

For *in vivo* experiments, PM2.5 was suspended in sterile water to a final concentration of 0.5 mg/mL and stored at 4 °C until use.

### 2.5 Histological analysis

Left lung tissues were fixed in 4% paraformaldehyde for 48 hours, embedded in paraffin, and sectioned at a thickness of 3 μm. Hematoxylin-eosin (HE) staining was performed for histopathological evaluation.

### 2.6 Lung histopathological scoring

Histological sections were examined under a light microscope at 100× magnification. Five random fields per section were assessed for inflammation severity using the ATS scoring criteria: a score of 1 indicates mild alveolar wall edema with limited inflammatory infiltration and hemorrhage involving less than 25% of the alveolar and interstitial spaces; a score of 2 reflects moderate inflammatory cell infiltration in both interstitial and partial alveolar spaces, with capillary congestion and edema affecting 25–50% of the lung tissue; a score of 3 denotes severe inflammation with dense infiltration and edema occupying 50–75% of the alveolar and interstitial areas.

### 2.7 Immunohistochemistry

Paraffin-embedded lung tissue sections were deparaffinized and rehydrated through a graded ethanol series. Antigen retrieval was performed, followed by quenching of endogenous peroxidase activity and blocking of nonspecific binding using 5% bovine serum albumin (BSA). The sections were then incubated overnight at 4°C with primary antibodies against TLR4 and NF-κB (both diluted 1:100). Signal detection was carried out using a horseradish peroxidase-linked polymer detection system. Hematoxylin counterstaining was applied to visualize nuclei.

### 2.8 Enzyme-linked immunosorbent assay

Bronchoalveolar lavage fluid (BALF) and cell culture supernatants were centrifuged at 1,000 rpm for 10 minutes, and the resulting supernatants were stored at −20°C until analysis. The concentrations of interleukin-1β (IL-1β) and IL-18 in both BALF and cell supernatants were quantified using commercially available ELISA kits (IL-18 from MultiSciences, Hangzhou, China; IL-1β from FineBiotech, Wuhan, China), following the manufacturers’ instructions.

### 2.9 Western blot analysis

Lung tissues and cultured cells were lysed in RIPA buffer supplemented with protease and phosphatase inhibitors. Lysates were centrifuged at 12,000 rpm for 30 minutes at 4°C, and the supernatants were collected. Protein concentrations were determined using a bicinchoninic acid (BCA) assay. Equal amounts of protein samples were separated by 10–12% SDS-PAGE and subsequently transferred onto polyvinylidene difluoride (PVDF) membranes (Millipore, MA, USA). Membranes were probed with primary antibodies against TLR4 (1:500), MUC1 (1:1000), IRAK4 (1:1000), and phosphorylated IRAK4 (p-IRAK4; 1:1000). Protein bands were visualized using enhanced chemiluminescence (ECL; Cell Signaling Technology, USA), and band intensities were quantified using ImageJ software.

### 2.10 Quantitative real-time PCR

Total RNA was extracted from lung tissue and cultured cells using TRIzol reagent according to standard protocols. RNA concentration and purity were measured spectrophotometrically. Complementary DNA (cDNA) was synthesized from purified RNA, and quantitative real-time PCR was conducted using SYBR Green PCR Master Mix (Solarbio, China). Expression levels of NLRP3, ASC, and Caspase-1 mRNA were analyzed. Specific primer sequences for target genes were designed and validated prior to use (**Table 1**).

**Table 1:** The sequences of the primer sets.

Gene	Primer direction	Sequence (5’ → 3’)
ASC	Forward	CTCAGGGCACAGCCAGAACAG
	Reverse	GCCATACAGAGCATCCAGCAA
Caspase-1	Forward	AGTGTAGGGACAATAAATGG
	Reverse	CTGATGGACCTGACTGAAGC
NLRP3	Forward	AGCCTTGAAGAGGAGTGGAT
	Reverse	TGGGTGTAGCGTCTGTTGAG
β-actin	Forward	GGCACCCAGCACAATGAA
	Reverse	TAGAAGCATTTGCGGTGG

### 2.11 Immunofluorescence staining

Human bronchial epithelial cells (16HBE) were seeded onto sterile glass coverslips and subjected to experimental treatments as indicated. After incubation, cells were washed with pre-warmed phosphate-buffered saline (PBS, 37°C) and permeabilized with 0.1% Triton X-100 (Beyotime, China) for 30 minutes. Following three PBS washes, nonspecific binding sites were blocked using 1% BSA for 1 hour at room temperature. Cells were then incubated overnight at 4°C with primary antibodies against NF-κB, GSDMD, NLRP3, ASC, and Caspase-1 (all at 1:100 dilution). Subsequently, Cy3- or FITC-conjugated goat anti-mouse/rat IgG (1:200 dilution) secondary antibodies were applied for 1 hour at room temperature. Nuclear counterstaining was performed using DAPI (Aladdin, China) for 5 minutes. After three additional PBS washes, slides were mounted using an anti-fade mounting medium and examined under a fluorescence microscope. Image analysis was conducted using Image-Pro Plus 6.0 software.

### 2.12 Flow cytometric analysis of pyroptosis

To assess cell pyroptosis, 16HBE cells were seeded into six-well plates (2 × 10^5^ cells/well) and exposed to PM2.5 suspension (0.5 mg/mL) for 24 hours. Following treatment, pyroptotic cell populations were quantified using an Annexin V/propidium iodide (PI) double-staining kit (Yeasen, China) and analyzed by flow cytometry.

### 2.13 DAPI/PI co-staining

For morphological observation of nuclear and membrane integrity, 16HBE cells (2 × 10^5^ cells/well) were treated with PM2.5 (0.5 mg/mL) for 24 hours. After removing the supernatant, cells were washed with PBS and fixed with 0.5 mL of fixative at room temperature for 20 minutes. Following triple PBS washes, DAPI (1:1000) and PI (1:200) staining solutions were applied sequentially. After a final PBS rinse, cells were visualized under a fluorescence microscope.

### 2.14 Measurement of caspase-1 activity

Caspase-1 enzymatic activity in cell lysates was determined using a colorimetric assay kit (Beyotime, China), following the manufacturer’s instructions. The absorbance of the reaction products was measured at 405 nm to assess relative enzyme activity.

### 2.15 Observation of cell morphology via phase-contrast microscopy

16HBE cells were cultured in six-well plates (2 × 10^5^ cells/well) and treated with PM2.5 suspension (0.5 mg/mL) for 24 hours. After discarding the supernatant and rinsing with PBS, cell morphology was evaluated under a phase-contrast microscope at 200× and 400× magnification.

### 2.16 Statistical analysis

Data analysis was conducted using SPSS version 20.0. Normality of distribution was assessed prior to statistical testing. Data conforming to normal distribution and homogeneity of variance were expressed as mean ± standard deviation (mean ± SD). For normally distributed data, one-way analysis of variance was used for group comparisons. If homogeneity of variance was met, the least significant difference test was applied for pairwise comparisons; if not, Dunnett’s T3 test was used for correction. For non-normally distributed data or data with unequal variances, the Kruskal-Wallis nonparametric test was used. A *p*-value < 0.05 was considered statistically significant.

The sample sizes for animal experiments (n=6 per group) and the number of replicates for cell-based assays (n=3) were chosen based on prior experience in similar experimental systems (8, 20, 21) and are consistent with established practices in the field. These parameters provided sufficient statistical power to detect significant intergroup differences while adhering to the 3R principles (Replacement, Reduction, Refinement) of animal welfare.

## 3 Results

### 3.1 MUC1 attenuates PM2.5-induced airway injury and inflammation

**Figure 1:**
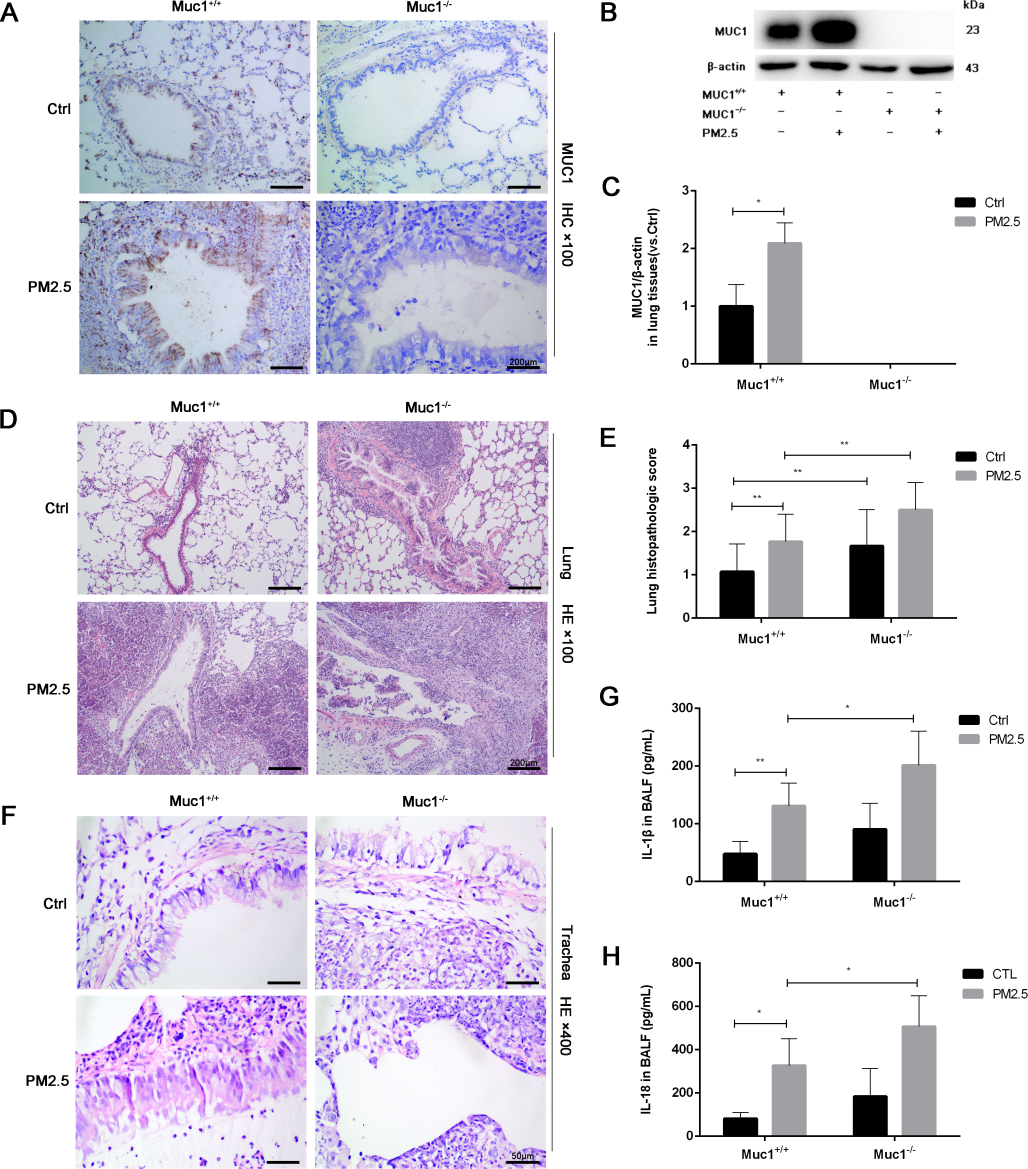
MUC1 attenuated PM2.5-induced airway injury and inflammation in rats. After 5 times of Saline/PM2.5 induction, rats were euthanized, and lung tissues were collected. **(A)** Immunohistochemical staining for MUC1 was perfomed in the Lung. Scale bars, 200 μm. **(B, C)** The protein levels of p-IRAK4 and IRAK4 was analyzed by Western blot. **(D)** Lung pathology was determined by HE staining with 100×. Scale bars, 200 μm. **(E)** Lung histopathological score was assessed. **(F)** Trachea pathology was determined by HE staining with 400×. Scale bars, 50 μm. Measurement of BALF levels of **(G)** IL-1β and **(H)** IL-18 via ELISA kits. Ctrl means Ctrl-Muc1^+/+^group. Data are presented as Mean ± SD, n = 6, ^*^ *P* < 0.05 and ^**^ *P* < 0.01.

To investigate the role of MUC1 in airway inflammation, we first examined its expression and localization across experimental groups. As expected, no MUC1 expression was detected in the lung tissues or airway epithelium of Muc1^−/−^ rats in either the control or PM2.5-treated groups. In contrast, MUC1 expression was significantly elevated in the lung tissues and airway epithelium of PM2.5-exposed Muc1^+/+^ rats compared to control Muc1^+/+^ rats (**Figures 1A–C**).

Histopathological analysis further clarified the protective role of MUC1 against PM2.5-induced airway injury. HE staining revealed that in the control Muc1^+/+^ group, the bronchiolar walls remained thin, with limited peribronchial inflammatory cell infiltration and intact alveolar septa. In contrast, the control Muc1^−/−^ group exhibited thickened bronchiolar walls, extensive peribronchial infiltration of inflammatory cells, interstitial fibrosis, and hyperplasia. PM2.5 exposure in Muc1^+/+^ rats led to bronchiolar wall thinning, substantial neutrophil infiltration, epithelial necrosis, and interstitial edema with alveolar septal thickening and rupture. These effects were more severe in PM2.5-exposed Muc1^−/−^ rats, which showed prominent fibrotic changes, intense inflammatory infiltration, necrotic exudates, granulation tissue formation, and loss of normal alveolar architecture (**Figure 1D**).

Airway-specific histological observations corroborated these findings. In the control Muc1^+/+^ group, the pseudostratified ciliated columnar epithelium was largely intact, with moderate goblet cell presence, a clear smooth muscle layer, and minimal inflammatory infiltration. The control Muc1^−/−^ group displayed moderate to severe submucosal fibrosis and increased infiltration by neutrophils, eosinophils, and lymphocytes. PM2.5-treated Muc1^+/+^ rats demonstrated partial epithelial disruption, serous exudates, and moderate fibrotic changes with occasional lymphocyte infiltration. In contrast, PM2.5-treated Muc1^−/−^ rats exhibited severe epithelial loss, goblet cell depletion, extensive inflammatory infiltration, and pronounced submucosal fibrosis with signs of cellular necrosis (**Figure 1F**). Quantitative scoring confirmed significantly higher lung injury scores in the PM2.5-Muc1^−/−^ group compared to the PM2.5-Muc1^+/+^ group (**Figure 1E**).

Moreover, ELISA results revealed significantly elevated levels of proinflammatory cytokines IL-1β and IL-18 in the bronchoalveolar lavage fluid of PM2.5-Muc1^−/−^ rats relative to the PM2.5-Muc1^+/+^ group (**Figures 1G, H**). Collectively, these findings demonstrate that MUC1 plays a protective role in mitigating PM2.5-induced airway inflammation and structural damage by reducing inflammatory cell infiltration, preserving epithelial integrity, and suppressing cytokine expression.

### 3.2 MUC1 regulates the IRAK4/NF-κB signaling axis in PM2.5-induced airway inflammation

Previous studies have implicated the TLR4/NF-κB signaling cascade as a key pathway in PM2.5-induced inflammation, partly through its upstream regulation of the NLRP3 inflammasome. Upon activation, this multiprotein complex facilitates the cleavage of pro-Caspase-1 into its active form, which subsequently promotes the secretion of IL-1β and IL-18, amplifying the inflammatory response (22).

**Figure 2:**
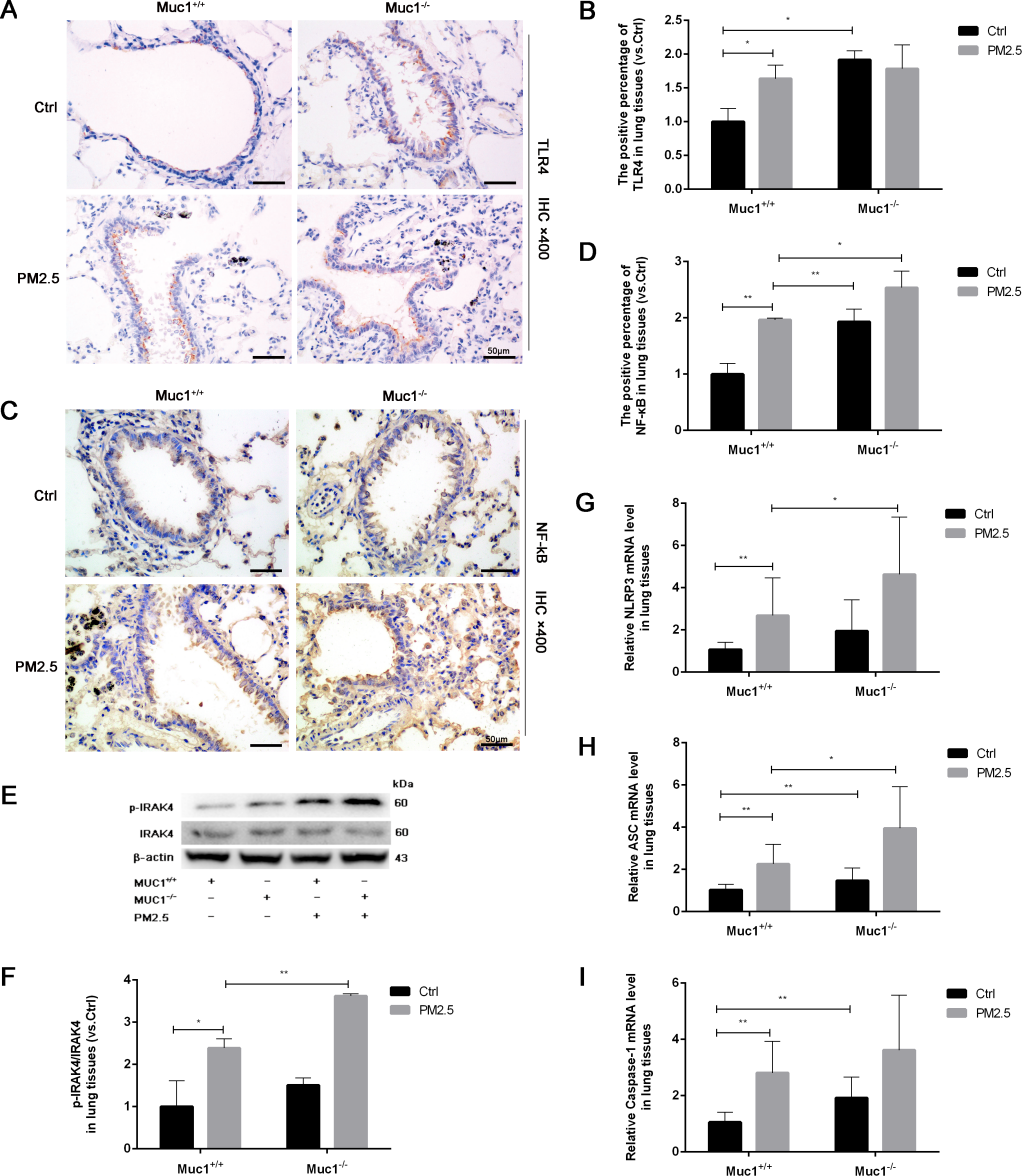
MUC1 regulates IRAK4/NF-κB signaling axis and affects PM2.5- induced airway inflammation in rats. **(A)** Immunohistochemical staining for TLR4 was perfomed in the Trachea. Scale bars, 50 μm. **(B)** The positive percentage of TLR4 in lung tissues was measured by Image J software. **(C)** Immunohistochemical staining for NF-κB was perfomed in the Trachea. Scale bars, 50 μm. **(D)** The positive percentage of NF-κB in lung tissues was measured by Image J software. **(E, F)** The protein levels of p-IRAK4 and IRAK4 was analyzed by Western blot. **(G-I)** The mRNA levels of NLRP3, ASC and Caspase-1 in the lung tissues were analyzed by Real-Time RCR. Ctrl means Ctrl-Muc1^+/+^group. Data are presented as Mean ± SD, n = 6, ^*^ *P* < 0.05 and ^**^ *P* < 0.01.

To explore whether MUC1 modulates this pathway, we performed immunohistochemical analysis of TLR4 and NF-κB in lung tissue. While TLR4 expression showed no significant difference between groups, NF-κB expression was markedly upregulated in the PM2.5-Muc1^−/−^ group compared to the PM2.5-Muc1^+/+^ group (**Figures 2A–D**). Western blotting further confirmed elevated phosphorylation of IRAK4 in Muc1-deficient rats exposed to PM2.5, indicating enhanced activation of the IRAK4/NF-κB axis in the absence of MUC1 (**Figures 2E, F**). These findings elucidate a key mechanism by demonstrating that MUC1 deficiency potentiates IRAK4 hyperactivation in response to PM2.5, which mechanistically supports its role as an endogenous brake on IRAK4 phosphorylation.

To assess downstream effects, RT-qPCR was used to quantify mRNA expression levels of NLRP3 inflammasome components. The results showed significant increases in NLRP3, ASC, and Caspase-1 mRNA in lung tissues from the PM2.5-Muc1^−/−^ group compared with their Muc1^+/+^ counterparts (**Figures 2G–I**). These data suggest that MUC1 suppresses PM2.5-induced activation of the TLR4/NF-κB signaling axis, thereby attenuating NLRP3 inflammasome assembly and subsequent inflammatory cytokine release. Thus, MUC1 appears to exert its anti-inflammatory effects by modulating key molecular mediators involved in PM2.5-triggered airway inflammation.

### 3.3 MUC1 modulates the IRAK4/NF-κB signaling axis and reduces PM2.5-induced inflammatory cytokine production in airway epithelial cells

To investigate whether MUC1 regulates PM2.5-induced inflammation via the IRAK4/NF-κB signaling pathway, 16HBE cells were transfected with a MUC1 overexpression plasmid or empty vector, with or without IRAK4 inhibitor treatment.

**Figure 3:**
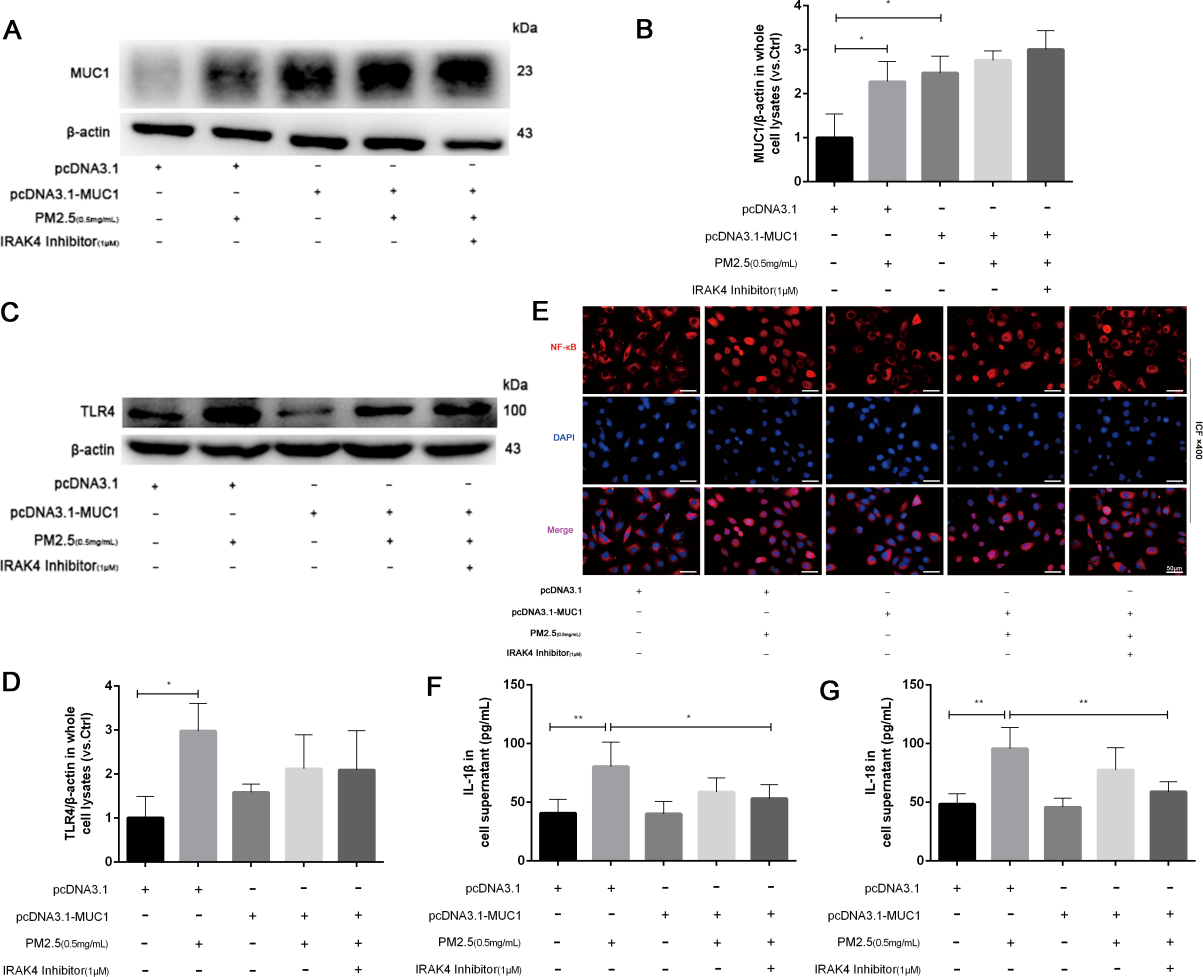
MUC1 regulates IRAK4/NF-κB signaling axis and reduces the release of 16HBE inflammatory cytokines induced by PM2.5. **(A)** The protein levels of MUC1 was analyzed by Western blot. **(B)** Densitometric analysis of proteins of interest in the immunoblots using β-actin as the internal reference. **(C)** The protein levels of TLR4 was analyzed by Western blot. **(D)** Densitometric analysis of proteins of interest in the immunoblots using β-actin as the internal reference. **(E)** Location alternation of NF-κB in cells was visualized through immunofluorescence (ICF) staining. Scale bars, 50 μm. **(F, G)** Measurement of Cell supernatant levels of IL-1β and IL-18 via ELISA kits. Ctrl means [pcDNA3.1(+), pcDNA3.1-MUC1(-), PM2.5(-), IRAK4 Inhibitor(-)]. Data are presented as Mean ± SD, n = 3, ^*^ *P* < 0.05 and ^**^ *P* < 0.01.

We first verified the efficacy of our genetic manipulations. Western blot analysis revealed that MUC1 protein expression was substantially upregulated in cells transfected with the pcDNA3.1-MUC1 plasmid and downregulated in those receiving the sh-MUC1 construct, relative to their respective controls (**Figure 3A**). These data thus confirm successful modulation of MUC1 at the functional protein level. Following PM2.5 exposure, a significant increase in TLR4 protein expression and marked nuclear translocation of NF-κB were observed, along with elevated inflammatory cytokine production. While MUC1 overexpression did not affect TLR4 expression levels, it did promote NF-κB nuclear translocation and cytokine release, suggesting that MUC1 alone can influence inflammatory signaling. However, pretreatment with an IRAK4 inhibitor significantly reduced NF-κB translocation and cytokine expression in both PM2.5-stimulated and MUC1-overexpressing cells (**Figures 3A–E**). These findings indicate that MUC1 may suppress PM2.5-induced inflammation primarily through inhibition of IRAK4 phosphorylation and downstream NF-κB activation, rather than through modulation of TLR4 expression. It is important to acknowledge that IRAK1/4 Inhibitor I is a dual inhibitor, and pharmacological interventions inherently present specificity limitations. Nevertheless, the specific role of the IRAK4/NF-κB axis in MUC1-mediated protection is strongly corroborated by a multi-faceted validation approach. Genetic experiments (both MUC1 overexpression and knockdown) yielded consistent functional data, and, crucially, the direct quantification of IRAK4 phosphorylation provides targeted biochemical evidence for the pathway’s engagement.

### 3.4 MUC1 inhibits PM2.5-induced pyroptosis of airway epithelial cells via the NLRP3/GSDMD pathway

**Figure 4:**
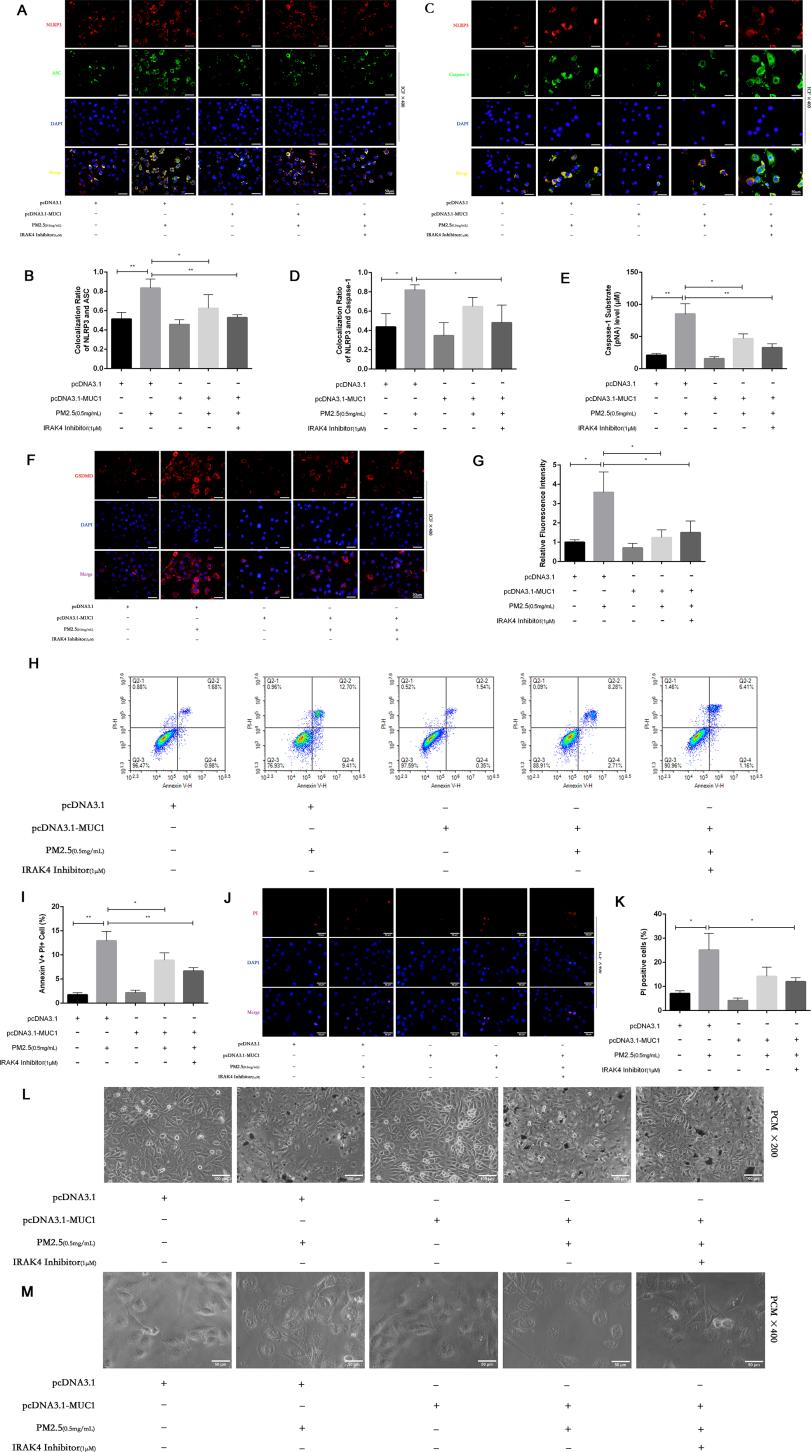
MUC1 inhibits pyrophosis of 16HBE cells induced by PM2.5 via NLRP3/GSDMD pathway. **(A, C)** Immunofluorescence staining for NLRP3/ASC and NLRP3/Caspase-1 was performed on 16HBE cells treated with and without PM2.5 for 24h. Scale bars, 50 μm. **(B)** Statistical chart of NLRP3 and ASC. **(D)** Statistical chart of NLRP3 and Capase-1. **(E)** Statistical chart of Caspase-1 substrate level. **(F)** Representative photomicrographs of GSDMD expression in 16HBE cells by Immunofluorescence, scale bar, 50 µm. **(G)** Immunofluorescence staining quantitative analysis were performed to detect the level of the GSDMD. **(H)** Flow cytometry assays were performed to show the cell pyroptosis. **(I)** Statistical analysis was used to show cell pyrophosis. **(J)** PI staining was used to observe cell pyroptosis under fluorescence microscopy. Scale bars, 50 μm. **(K)** Statistical chart of PI positive cells. **(L)** Cellular morphology was examined under phase contrast microscope (PCM), Scale bars, 100 μm. **(M)** Cellular pyroptosis phenotype was examined under phase contrast microscope, Scale bars, 50 μm. Ctrl means [pcDNA3.1(+), pcDNA3.1-MUC1(-), PM2.5(-), IRAK4 Inhibitor(-)]. Data are presented as Mean ± SD, n = 3, ^*^ *P* < 0.05 and ^**^ *P* < 0.01.

Pyroptosis is a form of programmed cell death mediated by inflammasome activation and Caspase-1–dependent cleavage of gasdermin family members, notably GSDMD. This process leads to pore formation in the cell membrane and ultimately results in cellular swelling, membrane rupture, and release of proinflammatory contents (23). Morphological assessment under light microscopy showed that PM2.5 exposure caused characteristic pyroptotic changes in 16HBE cells, including decreased refractive index, cell swelling, and membrane lysis. In contrast, MUC1-overexpressing cells maintained relatively intact morphology, exhibiting only mild swelling and brightness under the same conditions (**Figures 4L–M**).

PI staining further confirmed these observations. A marked increase in PI-positive cells was seen after PM2.5 treatment, indicating compromised membrane integrity (24). However, MUC1-overexpressing cells showed significantly fewer PI-positive cells, suggesting protective effects on membrane stability (**Figures 4J, K**). Annexin V/PI dual staining followed by flow cytometry revealed a reduction in Annexin V^+^PI^+^ (pyroptotic) cells in the MUC1-overexpressing group compared to vector control cells following PM2.5 exposure, supporting the conclusion that MUC1 attenuates pyroptosis (25) (**Figures 4H, I**).

To elucidate the underlying mechanism, immunofluorescence staining and Caspase-1 activity assays were performed. Results showed that MUC1 overexpression markedly reduced GSDMD expression, co-localization of the NLRP3 inflammasome complex, and Caspase-1 activation in PM2.5-exposed cells compared with controls (**Figures 4A–G**). These data collectively indicate that MUC1 inhibits PM2.5-induced pyroptosis in airway epithelial cells by downregulating the NLRP3/GSDMD signaling pathway.

The induction of pyroptosis is substantiated by a combination of assays, demonstrating caspase-1 activation, GSDMD immunofluorescence, loss of membrane integrity (via PI staining and flow cytometry), and characteristic morphological alterations. Although direct confirmation of GSDMD cleavage and LDH release would further solidify the mechanism, the collective agreement from these diverse methodological approaches offers robust, corroborative evidence for pyroptosis in our model.

### 3.5 Knockdown of MUC1 enhances TLR4/NF-κB activation and aggravates PM2.5-induced inflammatory responses in 16HBE cells

**Figure 5:**
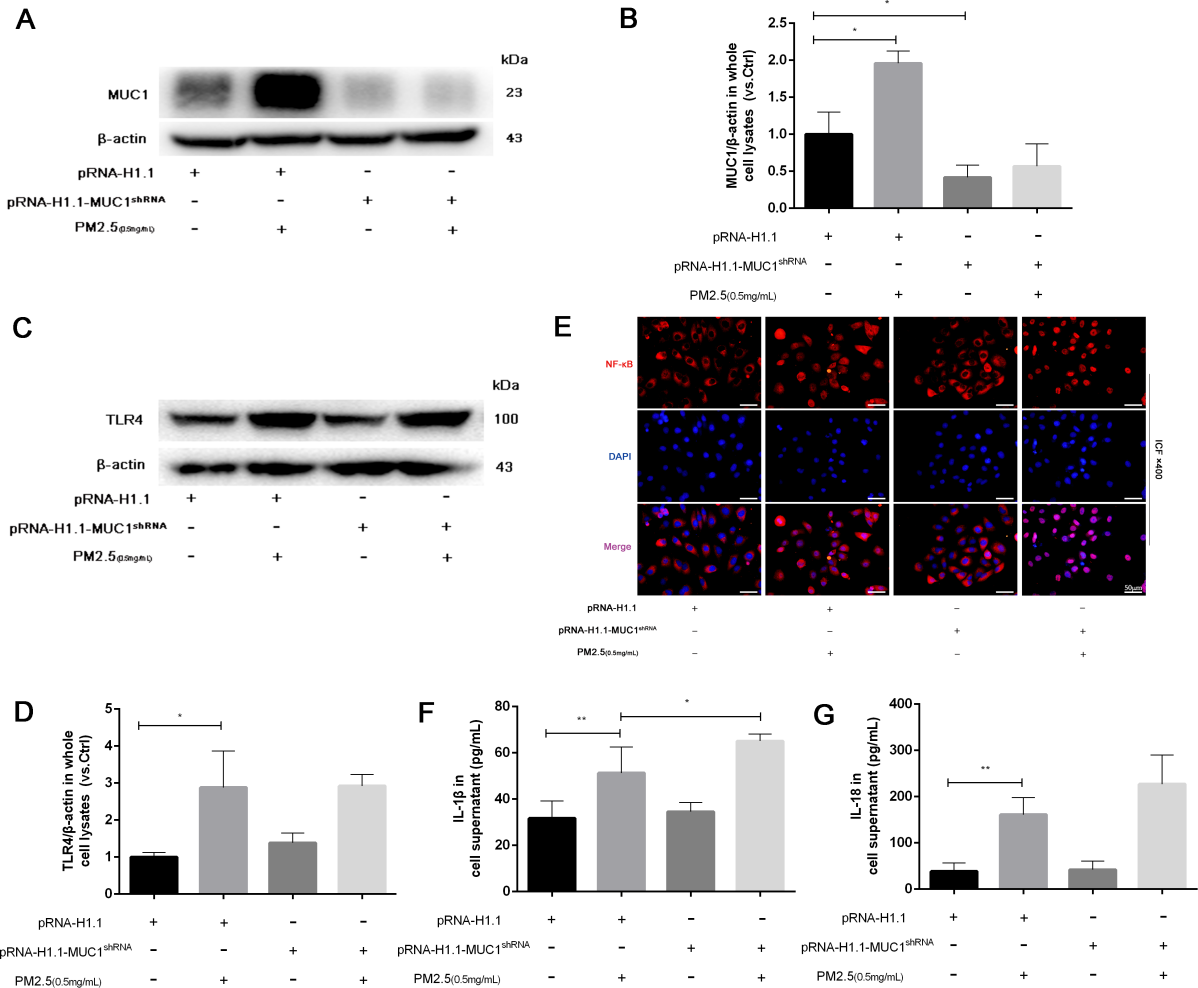
Knockdown of MUC1 promotes the activation of TLR4/NF-κB pathway and increases PM2.5-stimulated the inflammatory responses of 16HBE cells. **(A)** The protein levels of MUC1 was analyzed by Western blot. **(B)** Densitometric analysis of proteins of interest in the immunoblots using β-actin as the internal reference. **(C)** The protein levels of TLR4 was analyzed by Western blot. **(D)** Densitometric analysis of proteins of interest in the immunoblots using β-actin as the internal reference. **(E)** Location alternation of NF-κB in cells was visualized through immunofluorescence staining. Scale bars, 50 μm. **(F, G)** Measurement of Cell supernatant levels of IL-1β and IL-18 via ELISA kits. Ctrl means [pRNA-H1.1(+), pRNA-H1.1^shRNA^(-), PM2.5(-)]. Data are presented as Mean ± SD, n = 3, ^*^ *P* < 0.05 and ^**^ *P* < 0.01.

To further validate the regulatory role of MUC1 in inflammatory signaling, 16HBE cells were transfected with MUC1-specific knockdown plasmids or control vectors. Western blot analysis verified a drastic decrease in MUC1 protein expression following sh-MUC1 transfection, confirming the efficiency of the knockdown (**Figure 5A**). Following PM2.5 stimulation, TLR4 protein levels were not significantly altered by MUC1 knockdown; however, nuclear translocation of NF-κB was markedly enhanced, and inflammatory cytokine expression was significantly elevated (**Figures 5A–G**). These findings suggest that MUC1 acts downstream or parallel to TLR4, exerting its anti-inflammatory effects by suppressing NF-κB activation.

### 3.6 MUC1 knockdown promotes PM2.5-induced pyroptosis via activation of the NLRP3/GSDMD pathway

**Figure 6:**
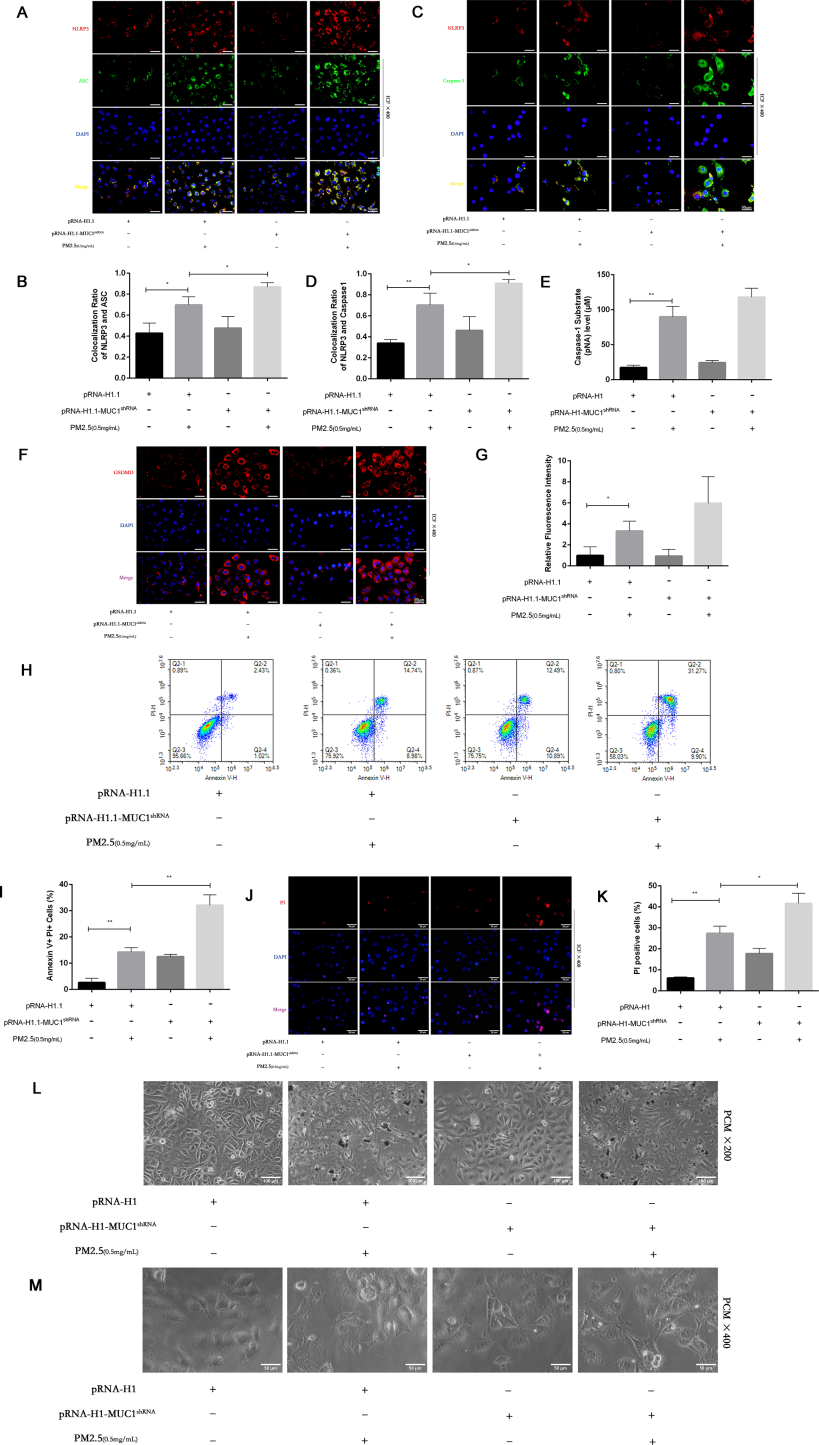
Effects of knockdown MUC1 on pyroptosis of PM2.5 stimulated 16HBE induced by NLRP3/GSDMD pathway. **(A, C)** Immunofluorescence staining for NLRP3/ASC and NLRP3/Caspase-1 was performed on 16HBE cells treated with and without PM2.5 for 24h. Scale bars, 50 μm. **(B)** Statistical chart of NLRP3 and ASC. **(D)** Statistical chart of NLRP3 and Capase-1. **(E)** Statistical chart of Caspase-1 substrate level. **(F)** Representative photomicrographs of GSDMD expression in 16HBE cells by Immunofluorescence, scale bar, 50 µm. **(G)** Immunofluorescence staining quantitative analysis were performed to detect the level of the GSDMD. **(H)** Flow cytometry assays were performed to show the cell pyroptosis. **(I)** Statistical analysis was used to show cell pyrophosis. **(J)** PI staining was used to observe cell pyroptosis under fluorescence microscopy. Scale bars, 50 μm. **(K)** Statistical chart of PI positive cells. **(L)** Cellular morphology was examined under phase contrast microscope, Scale bars, 100 μm. **(M)** Cellular pyroptosis phenotype was examined under phase contrast microscope, Scale bars, 50 μm. Ctrl means [pRNA-H1.1(+), pRNA-H1.1^shRNA^(-), PM2.5(-)].Data are presented as Mean ± SD, n = 3, ^*^ *P* < 0.05 and ^**^ *P* < 0.01.

To assess the impact of MUC1 deficiency on PM2.5-induced pyroptosis, we evaluated cellular morphology, PI staining, and Annexin V/PI flow cytometry in MUC1-knockdown 16HBE cells. Compared to control cells, MUC1-deficient cells displayed increased PI-positive and Annexin V^+^PI^+^ populations following PM2.5 exposure. Morphologically, these cells exhibited decreased refractivity, increased brightness, and typical features of pyroptosis including membrane swelling and rupture (**Figures 6H–M**).

Furthermore, immunofluorescence analysis and Caspase-1 assays demonstrated that MUC1 knockdown enhanced GSDMD expression, increased NLRP3 inflammasome co-localization, and promoted Caspase-1 activation in response to PM2.5 (**Figures 6A–G**). These results confirm that silencing MUC1 expression facilitates PM2.5-induced pyroptosis in airway epithelial cells through activation of the NLRP3/GSDMD axis.

## 4 Discussion

The present study provides novel insights into the regulatory role of MUC1 in airway inflammation and pyroptosis induced by PM2.5 exposure. The major findings can be summarized as follows: (1) PM2.5 induces airway inflammation and triggers pyroptotic cell death in bronchial epithelial cells; (2) MUC1 mitigates PM2.5-induced airway inflammation *in vivo*; (3) MUC1 suppresses PM2.5-induced inflammatory responses in 16HBE cells by inhibiting IRAK4 phosphorylation and downstream activation of the TLR4/IRAK4/NF-κB signaling pathway; (4) MUC1 attenuates PM2.5-induced pyroptosis through suppression of the TLR4/IRAK4/NF-κB axis and downstream activation of the NLRP3/GSDMD pathway. It is important to acknowledge that PM2.5-induced airway inflammation is a multifactorial process, involving intricate networks of signaling pathways and diverse cellular responses. While our study identifies a key protective function of MUC1 via the IRAK4/NF-κB/NLRP3 axis in airway epithelial cells, this mechanism operates within a broader pathological landscape. PM2.5-induced airway injury involves interconnected mechanisms, oxidative stress is recognized as a central player. ROS, for instance, are critical upstream activators of the NLRP3 inflammasome, a key pathway in this injury process. Although our previous research on DSQMT confirmed the importance of antioxidant effects (measured via ROS/MDA/SOD) in protection, the present study specifically focuses on elucidating the distinct role of MUC1 in regulating inflammatory signaling. Other processes—such as those associated with oxidative stress, autophagy, and additional pattern recognition receptors—are also likely to participate in the integrated inflammatory response to PM2.5 exposure.

It is well-established that PM2.5 exposure contributes to airway inflammation and the progression of various respiratory diseases. Although inflammation is a protective mechanism against external insults, persistent or excessive airway inflammation can lead to tissue damage. MUC1, a transmembrane glycoprotein predominantly expressed in bronchial epithelial cells, comprises an extracellular (EC) domain, a transmembrane segment, and a cytoplasmic tail (CT) (26). While the EC domain participates in mucosal barrier formation, the CT domain is involved in intracellular signaling. Beyond its widely studied role in tumor biology due to its aberrant glycosylation and overexpression in cancer, MUC1 has recently emerged as a critical endogenous regulator of inflammation. It has been shown to facilitate the resolution of inflammation during acute pulmonary infections (27). For instance, Muc1−/− mice exhibit exaggerated pulmonary inflammation and delayed recovery in response to Pseudomonas aeruginosa or Streptococcus pneumoniae infection compared with wild-type controls (28). *In vitro* studies also demonstrate that MUC1 silencing enhances cytokine production in bronchial epithelial cells challenged with respiratory syncytial virus or Haemophilus influenzae (29). Moreover, clinical studies have identified elevated levels of both the EC and CT domains of MUC1 in the sputum of patients during acute exacerbations of COPD (30, 31). These data support MUC1’s role as a key anti-inflammatory mediator in respiratory pathologies.

In the present study, we established a PM2.5-induced airway inflammation model using Muc1^+/+^ and Muc1^−/−^ rats, and complemented these findings with *in vitro* experiments using 16HBE cells transfected with MUC1 overexpression and knockdown constructs. Our results showed that Muc1^−/−^ rats exhibited more severe airway histopathological changes and greater inflammatory cell infiltration than Muc1^+/+^ rats following PM2.5 exposure. Similarly, MUC1 overexpression in 16HBE cells attenuated, whereas MUC1 silencing enhanced, the expression of proinflammatory cytokines. Collectively, these findings confirm that MUC1 functions as a suppressor of PM2.5-induced airway inflammation by limiting epithelial injury and downregulating inflammatory signaling.

One proposed mechanism underlying MUC1’s anti-inflammatory effect involves its interaction with toll-like receptors (TLRs). Upon pathogen recognition, TLRs, particularly TLR4, initiate signaling cascades that culminate in NF-κB activation and transcription of inflammatory genes (13). However, the CT domain of MUC1 has been reported to interfere with TLR ligand binding and downstream activation, thereby dampening the inflammatory response (32, 33). Our results are consistent with this notion: although TLR4 protein expression remained unchanged, NF-κB protein expression and nuclear translocation were significantly enhanced in MUC1-deficient rats and 16HBE cells following PM2.5 exposure. Conversely, MUC1 overexpression inhibited PM2.5-induced NF-κB activation. These findings suggest that MUC1 exerts anti-inflammatory effects by modulating the TLR4/NF-κB signaling axis. Future studies are warranted to precisely map the molecular targets within this axis that are regulated by MUC1 upon PM2.5 stimulation.

IRAK4 is a pivotal kinase in the TLR4/NF-κB pathway, serving as a proximal effector that undergoes autophosphorylation within the Myddosome complex. Activated IRAK4 subsequently phosphorylates IRAK1/2 and drives NF-κB-mediated transcription of proinflammatory cytokines (34). Previous research has shown that in gastric inflammation, MUC1 suppresses IRAK4 activity, thereby inhibiting NF-κB activation and subsequent transcription of inflammasome-related genes such as NLRP3 (16). In line with these findings, our study demonstrated that MUC1 overexpression or pharmacological inhibition of IRAK4 attenuated PM2.5-induced activation of NF-κB and downstream inflammasome components in 16HBE cells. These results highlight a key mechanistic role for MUC1 in controlling airway inflammation via inhibition of the IRAK4/NF-κB signaling axis. Our Western blot analysis of IRAK4 phosphorylation provides direct biochemical evidence that MUC1 suppresses its PM2.5-induced activation. Nevertheless, the precise mechanistic basis of this regulation—specifically, whether it involves a direct interaction or is mediated by adaptor proteins—remains an important subject for future investigation.

Pyroptosis, a proinflammatory form of programmed cell death, is mediated by inflammasome activation and subsequent Caspase-1 cleavage of GSDMD (35). Activation of the classical NLRP3 inflammasome pathway involves TLR4-mediated upregulation of NLRP3, ASC, and pro-inflammatory cytokines such as IL-1β and IL-18. Upon activation, NLRP3 forms a complex with ASC, which recruits and activates pro-Caspase-1. Cleaved Caspase-1 not only processes IL-1β/IL-18 but also cleaves GSDMD to execute pyroptosis, amplifying the inflammatory response (36). In COPD, baseline expression of NLRP3 and pro-IL-1β is elevated in stable phases, but full activation of inflammasomes typically occurs during acute exacerbations (37). Our study found that PM2.5 stimulation led to increased NLRP3, Caspase-1, and GSDMD activation, along with morphological features of pyroptosis such as cell swelling and membrane rupture. Notably, MUC1 overexpression inhibited these effects, while MUC1 knockdown enhanced them. These findings underscore the role of MUC1 in suppressing NLRP3-mediated pyroptosis through modulation of upstream inflammatory signaling.

In summary, the key contributions of this study include: (1) identification of MUC1 as a suppressor of PM2.5-induced airway inflammation; (2) demonstration that MUC1 attenuates inflammatory signaling via the TLR4/IRAK4/NF-κB axis; and (3) elucidation of MUC1’s role in inhibiting pyroptosis through downregulation of the NLRP3/GSDMD pathway. Future investigations will focus on characterizing the transcriptional control of NLRP3 by NF-κB and delineating the molecular interaction between MUC1 and IRAK4.

### 4.1 Limitations

Despite the valuable insights gained from this study, several limitations should be acknowledged. First, the pathogenesis of PM2.5-induced airway inflammation is inherently complex and likely involves multiple overlapping signaling pathways and cellular mechanisms. Although our results position MUC1 as a key regulator, its anti-inflammatory functions likely extend beyond a single molecular target. While our findings establish MUC1 as a significant endogenous regulator in PM2.5-induced airway inflammation, we recognize that comprehensive omics-level analyses or comparative pathway profiling would be required to fully delineate its relative contribution within the multifactorial inflammatory network. Such systematic investigations could further elucidate potential crosstalk between MUC1-mediated signaling and other inflammatory pathways activated by PM2.5 components.

Second, our study primarily focused on the TLR4/IRAK4/NF-κB signaling axis; other inflammatory pathways and cellular phenotypes potentially involved in the PM2.5-induced response, such as oxidative stress-related or other TLR-mediated pathways, were not examined. Future studies should investigate the interplay between MUC1 and oxidative stress pathways, as well as its potential crosstalk with other pattern recognition receptors, to establish a holistic model of its anti-inflammatory action against PM2.5.

Third, although we demonstrated that MUC1 suppresses IRAK4 activation, the precise molecular mechanism by which MUC1 interacts with or modulates IRAK4 remains undefined. Additionally, while we utilized a dual IRAK1/4 inhibitor as a pharmacological approach to corroborate our genetic data, this compound targets both IRAK1 and IRAK4, and the possibility of off-target effects cannot be entirely ruled out. Nevertheless, the consistent modulation of the signaling pathway observed across multiple experimental conditions, and the alignment between genetic interventions—both overexpression and knockdown of MUC1—and pharmacological suppression, reinforces the conclusion that MUC1 attenuates inflammation via the IRAK4/NF-κB signaling axis.

Fourth, regarding the evidence for pyroptosis, while we have provided multiple lines of evidence including caspase-1 activation, GSDMD expression, membrane integrity assays, and morphological assessment, we recognize that including specific pyroptosis inhibitors and direct measurement of GSDMD cleavage and LDH release would further strengthen our conclusions. Future studies incorporating these additional endpoints would provide a more comprehensive characterization of the pyroptotic process in this model.

Fifth, while our study provides comprehensive evidence for the cell-autonomous, anti-inflammatory role of MUC1 in airway epithelium, we did not measure systemic inflammatory markers, such as serum cytokines or systemic oxidative stress indicators. Although this work focused primarily on elucidating the protective function of MUC1 within the bronchial epithelium—the primary barrier against inhaled PM2.5—future studies that include systemic readouts would enable a more holistic understanding of how MUC1 may mitigate PM2.5-induced systemic inflammation.

Sixth, it is important to consider the cellular context of MUC1 expression. MUC1 is not confined to airway epithelial cells but is also present in various immune cells, such as alveolar macrophages. Consequently, the inflammatory phenotype observed in global Muc1−/− rats represents the integrated systemic consequence of MUC1 loss across multiple cell lineages. Our *in vitro* data from 16HBE bronchial epithelial cells, however, establish a cell-autonomous protective function for MUC1 within the epithelial compartment itself. To move beyond this integrated view and precisely attribute the pathophysiological contributions of MUC1, future research using cell type-specific knockout models—for instance, those based on the Cre-LoxP system—will be essential to delineate the distinct roles of epithelial and immune cell-specific MUC1 in PM2.5-induced airway inflammation.

Seventh, the characteristics of our *in vivo* exposure protocol should be taken into account. The intranasal PM2.5 instillation regimen administered on days 1, 7, and 14 models an acute-to-subacute exposure scenario. This approach successfully elicited marked airway inflammation and injury, thereby offering a reliable system for mechanistic exploration. However, it may not completely mirror the pathophysiology of chronic human respiratory conditions, such as asthma or COPD, which generally develop from sustained, low-level environmental exposure. Thus, while our results clarify key molecular mechanisms of PM2.5-triggered inflammation in acute and subacute phases, their applicability to chronic disease progression requires further validation through long-term exposure studies.

Finally, although our study offers detailed histopathological and molecular characterization of PM2.5-induced airway injury, we note the lack of direct functional measurements—such as respiratory mechanics or lung function parameters—in our animal model. While our previous work in this model system demonstrated impaired oxygenation, future investigations integrating comprehensive pulmonary function tests would help to better correlate our mechanistic insights with physiologically and clinically relevant functional outcomes.

Future investigations employing biochemical assays and interaction studies are necessary to elucidate the direct or indirect binding relationship between MUC1 and IRAK4, and to determine whether additional co-factors are involved in this regulatory process. Despite the absence of co-immunoprecipitation data confirming a physical MUC1-IRAK4 interaction, the consistent suppression of IRAK4 phosphorylation with MUC1 overexpression—and its augmentation with MUC1 knockdown—functionally validates that MUC1 modulates IRAK4 activation. To further validate the specificity of the IRAK4 pathway involvement, subsequent studies employing more selective IRAK4 inhibitors or performing rescue experiments with constitutively active IRAK4 would be beneficial. It should be noted that the 16HBE immortalized bronchial epithelial cell line was chosen for this investigation based on its well-established profile and high suitability for genetic modification; however, we recognize that immortalized lines may not entirely mirror the physiological behavior of primary human bronchial epithelial cells. Consequently, validating our principal findings in primary cell cultures will be essential to reinforce the clinical relevance of our conclusions and constitutes a critical objective for subsequent research. Furthermore, considering the documented role of Wnt5a in facilitating NF-κB activation and NLRP3 inflammasome assembly, we propose that Wnt5a may function as an upstream amplifier of the inflammatory cascade initiated by PM2.5. Whether MUC1 influences Wnt5a-triggered signaling or regulates Wnt5a expression itself remains an open question, meriting further investigation. Elucidating this interaction could deepen our understanding of the broader regulatory circuitry underlying PM2.5-induced airway inflammation.

## 5 Conclusion

In summary, this study demonstrates that MUC1 plays a protective role in attenuating airway inflammation induced by PM2.5 exposure. The underlying mechanism appears to involve the inhibition of the TLR4/IRAK4/NF-κB signaling pathway and the subsequent suppression of NLRP3/GSDMD-mediated pyroptosis in airway epithelial cells. These findings not only enhance our understanding of the molecular mechanisms contributing to PM2.5-induced airway injury but also identify MUC1 as a promising therapeutic target for the prevention and treatment of PM2.5-related respiratory diseases.

## Data Availability

The datasets presented in this study can be found in online repositories. The names of the repository/repositories and accession number(s) can be found in the article/**Supplementary Material**.
