# Supplementary figures and images for: MUC1 alleviates PM2.5-induced airway inflammation by inhibiting the IRAK4/NF-κB/NLRP3 mediated pyroptosis in airway epithelial cells
Source: Front Immunol. 2025 Oct 16;16:1653184. doi: 10.3389/fimmu.2025.1653184 (PMC12571608; doi:10.3389/fimmu.2025.1653184)

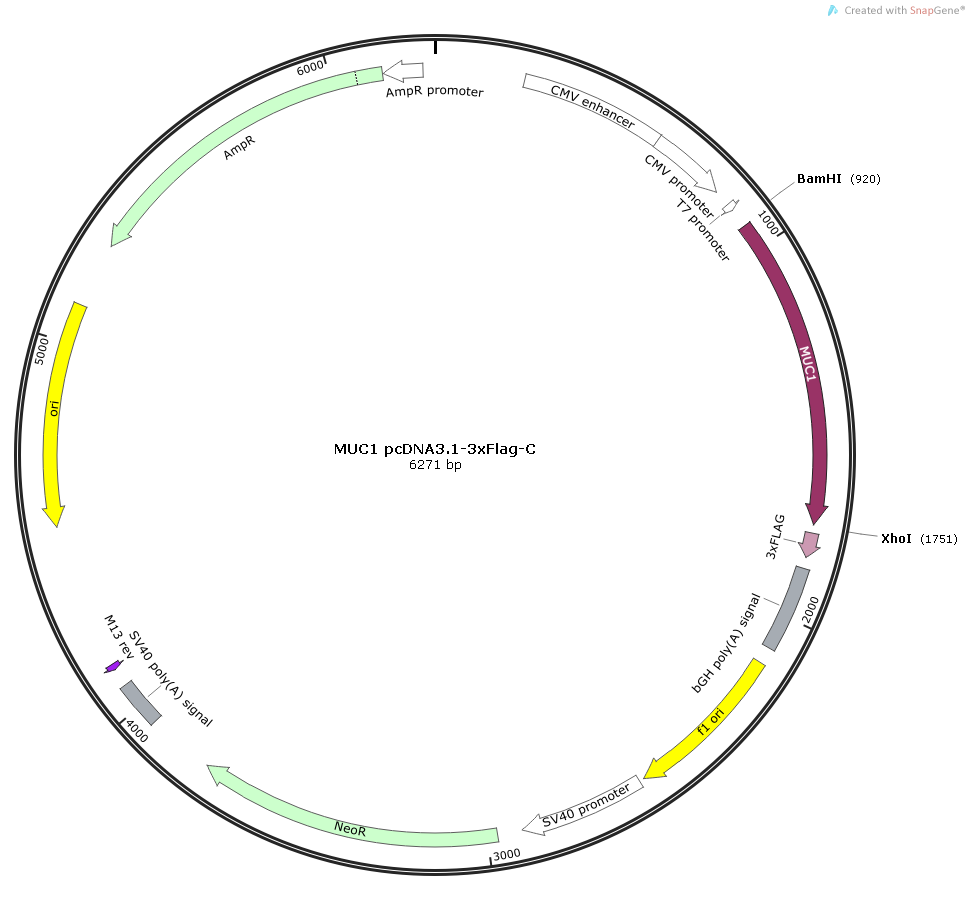

Supplement: Supplementary file 1 [file DataSheet1.zip › The datasets/pRNA-H1/MUC1 pcDNA3.1-3xFlag-C Map.png]
